# Association Between Social Support and Medication Literacy in Chinese Patients With Coronary Heart Disease

**DOI:** 10.3389/fcvm.2021.705783

**Published:** 2021-11-25

**Authors:** Li Qiao, Siqing Ding, Zhuqing Zhong, Xiaoqing Liu, Lin Lai, Feng Zheng

**Affiliations:** ^1^Department of Nursing, The Third Xiangya Hospital of Central South University, Xiangya School of Nursing, Central South University, Changsha, China; ^2^Department of Nursing, The Third Xiangya Hospital of Central South University, Changsha, China; ^3^Department of Cardiology, The Third Xiangya Hospital of Central South University, Changsha, China

**Keywords:** social support, medication literacy, association, coronary heart disease, patients

## Abstract

**Background:** The level of medication literacy is very important to control symptoms and improve the prognosis of patients with coronary heart disease (CHD). The positive role of social support is able to promote patient health outcomes. However, few studies have addressed the association between social support and medication literacy in patients with CHD. The purpose of this study is to investigate the status of medication literacy and social support, and confirm the association between them in patients with CHD.

**Methods:** This cross-sectional study investigated 416 participants, and was conducted in a grade a hospital in China. Three different survey instruments were applied: The Demographic Characteristics Questionnaire, the Chinese Version of the Medication Literacy Scale, and the Social Support Rating Scale. Pearson correlation analysis and ordinal logistic regression analysis were performed to analyze data.

**Results:** The results showed that the mean score of medication literacy among the 416 participants was 4.96 ± 4.68, 48.8% (203) participants with inadequate medication literacy. The independent determinants of medication literacy include gender, education level, course of disease, number of medicines, and subjective support in social support. The mean score of social support was 41.05 ± 6.16. The Pearson correlation analysis indicated that social support was positively correlated with medication literacy (*r* = 0.398, *P* < 0.01).

**Conclusion:** The study shows that the level of medication literacy and social support for patients with CHD are inadequate. Social support levels could have a positive effect on medication literacy of patients.

## Introduction

Coronary heart disease has become a chronic noncommunicable disease that seriously threatens human health. The 2018 Cardiovascular Diseases Report in China (1) estimated that 290 million Chinese people suffer from cardiovascular diseases, including 11 million suffering from coronary heart disease (CHD), accounting for the first cause of death (1). Although percutaneous coronary intervention can, at present, effectively save lives of patients with CHD, it cannot guarantee patient quality of life after discharge from hospital. The European Society of Cardiology recommends that patients with CHD should take antiplatelet drugs for a long time to reduce the rate of recurrence (2).

Timely, regular, and long-term adherence to medication can effectively improve symptoms and control further the development of the disease (3, 4). However, with the extension of discharge time for patients with CHD, the rate of medication utilization is lower, and patient medication compliance has decreased, which in turn affects health-related quality of life (5, 6). One study in China has shown that the rate of medication treatment in patients with CHD for anti-platelet drugs, β-blockers, angiotensin-converting enzyme inhibitors (ACEI)/angiotensin receptor antagonists (ARB), and statins was only 10.6%, 10.1%, 7.6%, and 1.4%, respectively (7). Nonadherence to medication will increase the risk of adverse cardiovascular events, such as cardiovascular mortality and cardiovascular hospitalizations (8). Nevertheless, medication compliance of CHD patients is closely related to medication literacy (9).

Medication literacy was first mentioned in 2005 as part of health literacy (10). Pouliot et al. (11) defined medication literacy as “the degree to which individuals can obtain, comprehend, communicate, calculate, and process patient-specific information about their medication to make informed medication and health decisions in order to take medication safely and effectively, and the delivered ways of the content is irrelevant (e.g., written, oral, and visual).” Good medication literacy can effectively improve adherence to lifestyle changes and medication in patients with CHD (12, 13). Patients with higher level of medication literacy usually demonstrate better medication compliance (14, 15). On the contrary, patients with low medication literacy may have problems in comprehending medication information and following medication compliance management instructions, such as incorrect medication-taking behavior, namely, untimely erratic medication taking and stopping medication on their own accord, which might contribute to diminished quality of life (15–17).

The level of medication literacy depends not only on individual factors but also on social factors. In the theory of social ecology, the behavior of taking medicine is considered to be a personal factor, while social support is considered to be a factor at the social level (18). Social support was proposed in 1976, and subsequently defined by numerous researchers based on the buffer model and the main effect model as “the total behavior of emotional support, information support and peer support” when an individual encounters obstacles who can obtain social support from relatives, friends, colleagues and religious group members, etc. through a certain social contact network (19–23). Social support is usually divided into three dimensions: subjective support, objective support, and utility of social support.

Social support can provide patients with material support, spiritual encouragement, and drug-related information support, which can correct the wrong medication concepts and methods of patients, and improve the knowledge of medication of patients. Due to a long course of disease, patients with CHD need lifelong medication, and they could be prone to experiencing negative emotions, such as anxiety and depression. Emotion support may mitigate the negative effects of emotions on patients with CHD (22, 24–26). Patients feel that they are respected, supported, and understood in society, and will deal with their medication problems with a more optimistic and positive attitude. Furthermore, researches reveal that social support can improve the ability of patients to obtain medical information. Patients with high utilization of social support will make full use of the social resources around them, obtain drug-related information through various channels, and improve their self-care management ability and medication compliance (27–29).

Currently, few studies are focused on the effect of social support on medication literacy and the relationship between medication literacy and social support in patients with CHD. Understanding the role that social support plays in medication literacy is essential in the development of medical standards of care practices. Therefore, we investigate the current situation and the correlation between medication literacy and social support in patients with CHD in order to provide a reference for the implementation of future medication literacy interventions for patients with CHD, and improve the level of medication literacy of the patients.

## Materials and Methods

### Study Design and Participants

This cross-sectional study was conducted between November 2018 and January 2019. The participants were recruited from the cardiology unit of a grade A hospital in Changsha, Hunan, China. Participant inclusion criteria included: (1) patients who had been diagnosed with CHD; (2) age of over 18 years with perfect cognition and communication ability; (3) currently taking at least one cardiovascular medication; and (4) agreed and volunteered to participate in this study. Exclusion criteria were: (1) people with medical work experience; (2) having a severe mental disorder; (3) patients with stage III or IV heart failure.

### Measures

#### Demographic Questionnaire

This study used a self-designed questionnaire to collect the demographic characteristics of the participants, such as gender, age, level of education, annual income, marital status, occupational status, coronary angiography results, type of medical insurance, course of disease, number of health problems, and number of medicines currently taken.

#### Chinese Version of the Medication Literacy Scale

This scale was originally developed in the United States by Sauceda et al. (30) at the University of Texas, Department of psychology. The scale is mainly used to measure the ability of patients to read, comprehend, calculate, and cope with medication-related problems in the medical information environment, in order to assess their level of medication literacy. The calculated value of the Kuder–Richardson Formula 20 (k-r20) is 0.81. After obtaining consent from the original scale author, Zheng et al. (31) translated and revised the scale into the Chinese version of the Medication Literacy Scale according to the national conditions in China. The Chinese version of the scale is a one-dimensional medication literacy assessment scale, and the retest reliability of the scale is 0.885. Fractional half-reliability was 0.840. K-R20 is 0.82. This scale provides four fictitious cases with a total of 14 items, which are scored on a two-point scale (1 for correct answers and 0 for errors); the higher the score, the higher the level of patient medication literacy. The full score for this scale is 14. According to the calculation formula principle of distinction degree, used in educational statistics, the scores were divided into three groups: adequate literacy (>10), marginal literacy (4–10), and inadequate literacy (<4). The Chinese version of the assessment of medication literacy has good reliability and validity, and can be used to evaluate medication literacy.

#### Social Support Rating Scale

This scale was compiled by Xiao (32) of the School of Public Health, Central South University, China. The scale is used to measure individual social relations, and includes three dimensions: objective support, subjective support, and utilization of social support, and contains a total of 10 items. The sum of the scores of items 2, 6, and 7 reflects objective support, which refers to the actual support objectively visible to the patient. The sum of the scores of items 1, 3, 4, and 5 reflects subjective support, which refers to the emotional support subjectively experienced. The sum of the scores of items 8, 9, and 10 reflects the utilization of social support, which refers to the utilization of support by individuals. The complete score is 66. Scores are categorized as follows: scores <22 are low level, scores 23–44 are medium level, and scores 45–66 are high level. Higher scores indicate better social support. The Cronbach's α of the total scale and three subscales is above 0.82, and internal consistency is 0.92, indicating good reliability and validity.

### Data Collection

The participants were required to complete questionnaires that included Demographic Questionnaire, Chinese Version of the Medication Literacy Scale, and Social Support Rating Scale. All the questionnaires were filled out by the participants, and collected by two trained researchers. For illiterate subjects, the researchers read the items word by word, and responses of the subjects were recorded on the questionnaires. Each patient completed the questionnaires within ~20 min. The questionnaires were gathered immediately after completion and checked for missing information by the researchers. In case of incomplete questionnaires, the patients were told to complete them as much as possible or the questionnaires would be regarded as invalid.

### Statistical Analysis

All data were analyzed by two researchers using the SPSS (version 19.0; IBM, Chicago, IL, United States.) software, and *P* < 0.05 was considered statistically significant. Sociodemographic and clinical characteristics of the sample was analyzed *via* descriptive statistics, such as, frequencies, percentages, and mean scores. A Pearson correlation analysis was conducted to identify the correlation between social support and medication literacy. With medication literacy as the outcome variable, an ordinal logistic regression analysis was performed to determine the predictors of medication literacy in patients with CHD.

## Results

### Sample Characteristics

In this study, 416 patients were ultimately eligible to participate. General demographic characteristics of the 416 participants are shown in Table 1. The majority of the participants were male (71.2%), married (91.3%), retired (45.9%), and have medical insurance (96.4%). The average age was 62.04 years [standard deviation (SD) = 11.25), with 63.2% older than 60 years old. Most of the participants had graduated from junior middle school or primary school (61.8%), and 65.4% had an annual income < ¥30,000. The mean (SDs) of the number of medicines that the patients were taking was 4.21 (SD = 2.72).

**Table 1:** Characteristics of patients (*N* = 416) with coronary heart disease, Changsha, Hunan, China.

**Items**	***n* (%)**
**Age (years)**	
<45	30 (7.2)
45–59	123 (29.6)
60–74	210 (50.5)
75–89	53 (12.7)
**Gender**	
Male	296 (71.2)
Female	120 (28.8)
**Education (years)**	
≤6	140 (33.7)
7–9	117 (28.1)
10–12	91 (21.9)
≥13	68 (16.3)
**Annual household income (in CNY)**	
<11,300	137 (32.9)
11,300–30,000	135 (32.5)
>30,000	144 (34.6)
**Marital status**	
Single	3 (0.7)
Married	380 (91.3)
Widowed	30 (7.2)
Divorced	3 (0.7)
**Employment**	
Employed	75 (18.0)
**Items**	***n*** **(%)**
Retired	191 (45.9)
Unemployed	118 (28.4)
Other	32 (7.7)
**Medical insurance**	
Urban medication insurance	220 (52.9)
Rural medication insurance	181 (43.5)
Self-paying	7 (1.7)
Others	8 (1.9)
**Coronary angiography results**	
Atherosclerosis of coronary artery	12 (2.9)
Single vessel disease	104 (25.0)
Double vessel disease	78 (18.8)
Triple vessel disease	222 (53.4)
**Disease course (years)**	
≤1	226 (54.3)
1–3	70 (16.8)
3–5	36 (8.7)
5–10	45 (10.8)
≥10	39 (9.4)
**Number of health problems**	
≤1	181 (43.5)
2	133 (32.0)
≥3	102 (24.5)
**Number of medicines currently taken**	
1	89 (21.4)
2–3	74 (17.8)
4–5	116 (27.9)
≥6	137 (32.9)

*CNY, Chinese Yuan*.

### Responses to the Chinese Medication Literacy Scale for Patients With CHD

The investigation results on each item of the medication literacy scale for patients with CHD are shown in Table 2. The mean medication literacy scores were 4.96 (SD = 4.68, range 0–14). Patients who had an adequate level of medication literacy (score >10) had a total of 82 (19.7%), and those who had a medium level of medication literacy (score 4–10) were 131 (31.5%), while 203 (48.8%) obtained a score <4, and were considered to have “inadequate medication literacy level.” The results showed that only 30.8% of the patients knew where to obtain new medication prescriptions. More than 70% of the patients did not understand the number of times that their prescribed drugs should be taken each day, nor did they know the dosage, injection site, or angle of injection. Only 35.3% of relatives were aware of the medication dosage that the patients should take. The item “Looking at this prescription, what is the name of the medicine that you need to buy at the pharmacy?” with a “Yes” answer accounted for 50.7%. In Case Scenario 4, 71.6% of the patients did not know medicine expiry date. Only 28.1% of the patients knew the reason why they should stop taking their medicine.

**Table 2:** Medication literacy of patients with coronary heart disease, Changsha, Hunan, China (*N* = 416).

**Item**		**Correct answers [*n* (%)]**
**Case Scenario 1**		
a1	According to the label, how many times one day should your mother inject the medicine?	106 (25.5)
a2	Please show me how much medicine you should put into the syringe in the morning, and mark on the Syringe.	111 (26.7)
a3	According to the instruction, please tell us or point out where the three parts of the body your mother could inject the medicine?	93 (22.4)
a4	According to the instruction, please tell me what is the right angle you should inject the medicine?	123 (29.6)
a5	Looking at the prescription, if your mother's medicine is run out, from whom you should get a new prescription?	128 (30.8)
**Case Scenario 2**		
a6	Looking at the instructions on this box, how much dosing of the medicine should you give to your niece?	96 (23.1)
a7	If you know the dosage of medicine that your niece needs to take, please mark down on the cup to what line you poured medicine.	181 (43.5)
a8	According to the directions, what is the maximum dosage should your niece take?	147 (35.3)
**Case Scenario 3**		
a9	Looking at this prescription, what is the name of the medicine that you need to buy at the pharmacy?	211 (50.7)
a10	According to the prescription, how many pills should you take?	198 (47.6)
a11	Looking at this bottle, the medicine in the bottle has the similar purpose with the medicine on the prescription, if you need to take 30 pills to cure the infection, how many boxes should you buy to have the correct amount of antibiotic required by the original prescription?	188 (45.2)
**Case Scenario 4**		
a12	Looking at the box, when does the medicine go out of date?	118 (28.4)
a13	According to the directions, what is or what are the active ingredients in each pill?	238 (57.2)
a14	Please look carefully of the box, for what reason should you stop the medicine?	117 (28.1)

### Responses to the Social Support Rating Scale

The score range of the scale was 0–66. The results showed that the mean score of social support was 41.05 (SD = 6.16). The mean scores of each dimension were 9.34 (SD = 2.5) for the objective support dimension, 24.41 (SD = 4.09) for the subjective support dimension, and 7.3 (SD = 2.49) for the support utility dimension. Responses for each dimension of the Social Support Rating Scale are summarized in Table 3.

**Table 3:** Scores for the overall level of social support and for each dimension (*n* = 416).

**Dimensions**	**Lowest score**	**Highest score**	**Mean**	**SD**
Objective support	3.00	18.00	9.34	2.50
Subjective support	15.00	32.00	24.41	4.09
Support utility	3.00	12.00	7.30	2.49
Total score	24.00	59.00	41.05	6.16

### Correlation Between Social Support and Medication Literacy

Table 4 shows the results of the correlation between medication literacy and social support. With medication literacy as the outcome variable, social support was positively correlated with medication literacy (*r* = 0.398, *P* < 0.01). The dimensions objective support (*r* = 0.252, *P* < 0.01) and subjective support (*r* = 0.477, *P* < 0.01) were positively associated with medication literacy. However, the dimension support utility was not found to be associated with medication literacy at a significant level.

**Table 4:** Correlation between social support and medication literacy of patients (n = 416) with coronary heart disease.

	**Objective support**	**Subjective support**	**Support utility**	**Total score of social support**
Medication literacy	0.252^**^	0.477^**^	0.029	0.398^**^

** *P < 0.01 were considered statistically significant (two-tailed)*.

### Predicting Factors of Medication Literacy

Variables of participant characteristics and three dimensions of social support, namely, objective support, subjective support, and support utility, were included in the ordinal logistic regression analysis as independent variables. Table 5 shows the results of the ordinal logistic regression analysis of medication literacy among patients with CHD. Five significant factors independently associated with medication literacy were as follows: gender [OR 0.607 (95% confidence interval, CI: −0.992, −0.006); *P* = 0.047], education level [OR 2.487 (95% CI: 1.07, 1.151); *P* < 0.001], course of disease [OR 1.21 (95% CI: 0.039, 0.344); *P* = 0.014], number of medicines [OR 0.78 (95% CI: −0.455, −0.041); *P* = 0.019], and subjective support [OR 1.07 (95% CI: 0.012, 0.124); *P* = 0.017]. With high level of education, smaller number of medicines, longer disease course, and subjective support, the medication literacy of patients with CHD increased, with a significant difference between males and females.

**Table 5:** Results of ordered logistic regression analysis of factors influencing medication literacy.

	**β**	**SE**	**Odds ratio**	**P**	**95% CI**
Gender	−0.499	0.252	0.607	0.047	(−0.992, −0.006)
Education level	0.911	0.123	2.487	<0.001	(1.070, 1.151)
Disease course	0.191	0.078	1.210	0.014	(0.039, 0.344)
Number of medicines	−0.248	0.105	0.780	0.019	(−0.455, −0.041)
Subjective support	0.068	0.028	1.070	0.017	(0.012, 0.124)

## Discussion

The results showed that the medication literacy score of 416 patients with CHD was 4.96 ± 4.68, which was lower than that in previous studies (9, 33). Of these patients, the results indicated that ~5 out of every 10 participants had low medication literacy. Low medication literacy was associated with several independent predictors, such as gender, education level, disease course, number of prescribed medicines, and subjective support. The results with consistent with previous studies (34, 35). Research (36) shows that elderly patients who are over 60 years old has the lowest level of medication literacy, which is related to the gradual decline of memory and understanding ability of the elderly as well as the weakening of their ability to accept new things. Therefore, family members should be encouraged to participate in the medication management of elderly patients to improve their medication literacy. In addition, our results indicated that patients with higher education level, taking a lower number of medicines, and with longer disease course were like to have higher medication literacy. The higher the educational level of patients, the stronger their ability to obtain medication information, the wider the channels for obtaining information, and the stronger the ability to self-manage, enabling patients to make correct medication decisions.

Notably, we found the medication literacy scores of female patients to be lower than those of male patients. The reason may be that the overall education levels of female patients in China are not as high as those of male patients (37), limiting the ability of female patients to obtain medication information, and leading to low levels of overall medication literacy. Considering the medication literacy scores, it is suggested that the levels of medication literacy had further room to improve.

In this study, the results (Table 3) showed that patient social support score (41.05 ± 6.61) was at a medium level. The scores on the dimensions subjective support, objective support, and utilization were 24.41 ± 4.09, 9.34 ± 2.5, and 7.3 ± 2.49, respectively. Subjective support refers to the emotional experience and satisfaction that a patient feels for being supported and understood in their social interactions, and is closely related to the subjective feelings of the patient (32). Objective support refers to the actual and visible help received by patients from family, spouse, friends, colleagues, etc., and includes both material and information support (32). Utility reflects patients seeking social support methods and approaches, as well as their degree of acceptance of social support (32). It can be seen that patients receive good emotional support during their illness, but that objective support and support utility need to be improved. Our findings revealed that patients themselves, as well as, their relatives, did not understand the frequency required for taking their medicine, nor were they aware of medication dosages, medication expiration dates, source of drugs, and reasons for discontinuing drug treatment. The results of this study are consistent with those of previous medical literacy surveys on patients with acute coronary artery disease (30).

Therefore, medical staff should encourage patients to participate in social activities to obtain information support and material support from relatives. Meanwhile, medical staff should assess patient acceptance of social support, with personalized social support provided to help them achieve their health goals. Strengthening social support of patients by the supervision of taking medication by relatives and health guidance from community health workers can redress incorrect concepts and behaviors of patients taking medication, as well as improve their medication literacy (38).

The results in Table 4 show a positive correlation between social support and patient medication literacy (*r* = 0.398, *P* < 0.01). It indicates that strengthening patient social support can, to some extent, improve patient medication literacy levels. Therefore, if patients receive more social support, patient medication literacy will improve, resulting in a positive effect on disease prognosis. Furthermore, the findings showed that two dimensions of social support, namely, subjective and objective support, had a significantly positive correlation with medication literacy, although the correlation was weak (*r* = 0.252, *P* < 0.01; *r* = 0.477, *P* < 0.01). Specifically, the results showed that only the subjective support dimension of social support was recognized as a factor affecting medication literacy in patients with CHD, according to the logistic regression analysis. This might be due to limited sample size in this research, although the sample size was based on correct calculation.

However, it is notable that the utilization of social support is not related to medication literacy. The result is not comparable with those of other research studies worldwide (23, 39). This may be related to the majority of the elderly over 60 years old in this study. The study (40) has found that with increasing age of the elderly, their physiological functions undergo degenerative changes, which leads to inconvenience, degeneration of cognitive functions, and slow behavioral responses, and restricts the social activities of the elderly, thereby preventing them from using social support to a certain extent of initiative. Considering this contradiction, further studies might be required to explain the interaction between support utility and medication literacy.

### Study Strengths and Limitations

A few strengths of this study are as follows: first, the results suggest that social support is associated with medication literacy, whereas few prior studies explored the relationship between social support and medication literacy. It is hoped that this study will encourage healthcare staff to place increased emphasis on social support when implementing measures to improve patient medication literacy. Second, validated assessments of the medication literacy scale and social support rating scale were performed in this study to ensure that data collection was valid.

This study also has some limitations: first, the study sample size was limited, and because of geographical restrictions, the study results cannot be extended to all patients with CHD. Therefore, we hope that future research would expand the sample size across regions and further explore the relationship between social support and medication literacy to verify the results of this study. Second, the questionnaires were self-reports; we lacked objective measures of social support and medication literacy. Thus, the reported social support levels might have been biased. However, this bias could have partly been decreased by the use of anonymous questionnaires.

## Conclusion

Our results show that the level of medication literacy and current social support for patients with CHD need to be improved, and that there is a positive correlation between social support and medication literacy. To improve patient medication literacy, care services should focus on strengthening social support function, building supportive environments, and connecting social support resources.

## Data Availability Statement

The raw data supporting the conclusions of this article will be made available by the authors, without undue reservation.

## Ethics Statement

The studies involving human participants were reviewed and approved by the Ethics Review Board of The Third Xiang Ya Hospital of Central South University (2018-S284). The patients/participants provided their written informed consent to participate in this study.

## Author Contributions

FZ conceived the study. SD, ZZ, XL, and LL collected, verified, and analyzed the data. FZ and LQ drafted the manuscript. All the authors provided critical revision of the manuscript for important intellectual content.

## Funding

This study was supported by the Natural Science Foundation Project of Hunan Province #1 under Grant No. 2019JJ50905 and China and the Health and Family Planning Commission Project of Hunan Province #2 under Grant No. B20180676.

## Conflict of Interest

The authors declare that the research was conducted in the absence of any commercial or financial relationships that could be construed as a potential conflict of interest.

## Publisher's Note

All claims expressed in this article are solely those of the authors and do not necessarily represent those of their affiliated organizations, or those of the publisher, the editors and the reviewers. Any product that may be evaluated in this article, or claim that may be made by its manufacturer, is not guaranteed or endorsed by the publisher.
